# Gender Bias in U.S. Pediatric Growth Hormone Treatment

**DOI:** 10.1038/srep11099

**Published:** 2015-06-09

**Authors:** Adda Grimberg, Lina Huerta-Saenz, Robert Grundmeier, Mark Jason Ramos, Susmita Pati, Andrew J. Cucchiara, Virginia A. Stallings

**Affiliations:** 1 Department of Pediatrics, Perelman School of Medicine, University of Pennsylvania, Philadelphia, PA; 2 Division of Endocrinology and Diabetes, The Children’s Hospital of Philadelphia, Philadelphia, PA; 3 Leonard Davis Institute of Health Economics, University of Pennsylvania, Philadelphia, PA; 4 Department of Pediatrics, Albert Einstein Medical Center, Philadelphia, PA; 5 Center for Biomedical Informatics, The Children’s Hospital of Philadelphia, Philadelphia, PA; 6 Clinical and Translational Research Center and Center for Clinical Epidemiology and Biostatistics, Perelman School of Medicine, University of Pennsylvania, Philadelphia, PA; 7 Division of Gastroenterology, Hepatology and Nutrition, The Children’s Hospital of Philadelphia.

## Abstract

Growth hormone (GH) treatment of idiopathic short stature (ISS), defined as height <−2.25 standard deviations (SD), is approved by U.S. FDA. This study determined the gender-specific prevalence of height <−2.25 SD in a pediatric primary care population, and compared it to demographics of U.S. pediatric GH recipients. Data were extracted from health records of all patients age 0.5–20 years with ≥ 1 recorded height measurement in 28 regional primary care practices and from the four U.S. GH registries. Height <−2.25 SD was modeled by multivariable logistic regression against gender and other characteristics. Of the 189,280 subjects, 2073 (1.1%) had height <−2.25 SD. No gender differences in prevalence of height <−2.25 SD or distribution of height Z-scores were found. In contrast, males comprised 74% of GH recipients for ISS and 66% for all indications. Short stature was associated (P < 0.0001) with history of prematurity, race/ethnicity, age and Medicaid insurance, and inversely related (P < 0.0001) with BMI Z-score. In conclusion, males outnumbered females almost 3:1 for ISS and 2:1 for all indications in U.S. pediatric GH registries despite no gender difference in height <−2.25 SD in a large primary care population. Treatment and/or referral bias was the likely cause of male predominance among GH recipients.

In 2003 the U.S. Food and Drug Administration (FDA) approved growth hormone (GH) treatment for children with idiopathic short stature (ISS), defined as height more than 2.25 standard deviations (SD) below mean for age and gender, and without evidence of underlying disease. This represents the shortest 1.2% of the U.S. population, using the 2000 Centers for Disease Control and Prevention [CDC] growth chart data. Prior to the FDA ruling, GH was prescribed primarily for GH deficiency, with an estimated prevalence of 1 in 3500 children^1^. Now about 1 in 100 children may be eligible for a treatment that requires daily subcutaneous injections and costs about $20,000 annually per patient^2^. The ISS indication shifted focus from underlying pathophysiology to stature as the criterion for treatment, with ramifications for health expenditures and policy considerations regarding the medicalization of a physical trait.

An international post-marketing registry of pediatric GH recipients showed that the 64% male predominance among U.S. patients exceeded the male:female proportions in other countries, and the greatest gender difference occurred for the familial short stature/constitutional growth delay/ISS group^3^. Also, males outnumbered females about 2:1 among short stature evaluations at a large U.S. pediatric endocrinology center^4^. Does this pattern of male predominance in subspecialty care and GH treatment reflect a greater prevalence of growth impairment in males or gender-based referral and/or treatment biases? In an urban, low income, primary care group, the prevalence of growth faltering did not differ by gender^5^. We now investigated the prevalence of short stature below the ISS threshold by gender in a large, heterogeneous pediatric primary care population. Additionally, this regional cohort was compared with treatment patterns in the GH registries for U.S. patients.

## Results

### Pediatric primary care population

Of the 189,280 subjects evaluated, 2073 (1.1%) had height <−2.25 SD, the FDA height criterion for ISS. Characteristics of the ISS and non-ISS groups were compared in Table 1. Except for gender, differences between the two groups were statistically significant (P < 0.0001) for all patient characteristics. Males comprised 51% of the ISS and 50% of the non-ISS groups (P = 0.35).

Height below the ISS threshold was modeled by logistic regression against patient characteristics among the 145,710 subjects with complete data (Table 2). Gender was not significant; male gender had an odds ratio (OR) of 0.96 (95% C.I. [0.87–1.06], P = 0.38). The factors significantly (P < 0.0001) associated with ISS-level height were, in descending value of ORs: history of prematurity, age, race/ethnicity (Hispanic and Asian >White >Black), Medicaid insurance, and BMI Z-score. Because prematurity had such a strong effect in the model, yet applies to distinct subjects, the analyses also were performed for the premature subgroup (n = 4,496) and non-premature subgroup (n = 141,214) separately (Table 3). Results were similar, except practice location was significant for the non-premature subgroup only (P = 0.02), and the racial/ethnic profiles varied slightly between the two subgroups. Of note, gender was not significant in either group.

**Figure 1:**
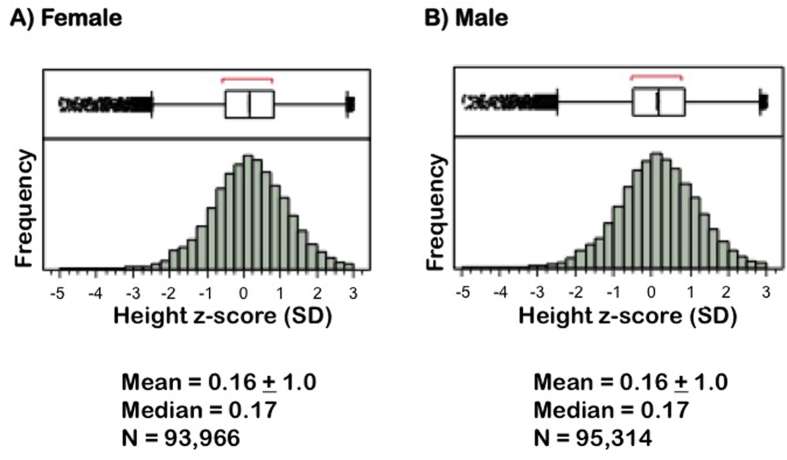
Distributions of height Z-scores by gender in the entire study population. Above each histogram is an outlier box plot; the boxes, demarcating the interquartile range and median, surround the middle half of the data points, while the tails extend to the farthest value still within 1.5 interquartile ranges from the quartiles. All data points beyond the tails are shown individually as outliers. Means are presented as ± SD.

**Figure 2:**
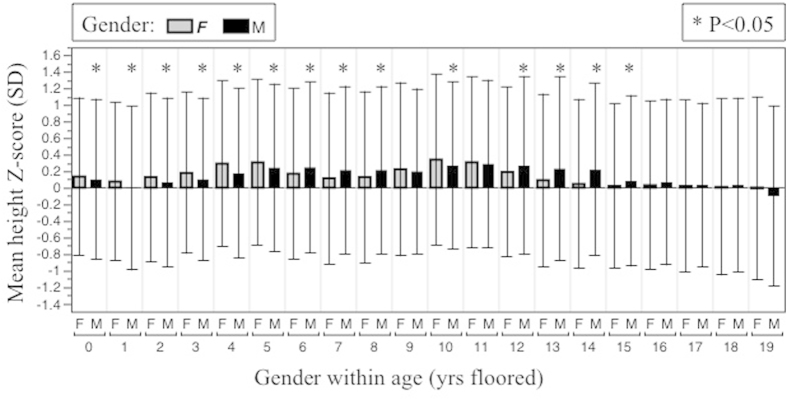
Age-specific distributions of height Z-scores by gender. Mean height Z-score ± 1 SD is shown for each gender for each year of age floored. Gender differences for each age were compared by one-way ANOVA.

Thus, gender was not a significant factor in the univariate or any of the multivariable analyses of ISS-level height. Further, distributions of height Z-scores by gender in the entire study population were found to be similar (Fig. 1). Comparison of age-specific mean height Z-scores by gender are shown in Fig. 2.

### GH registry data

**Figure 3:**
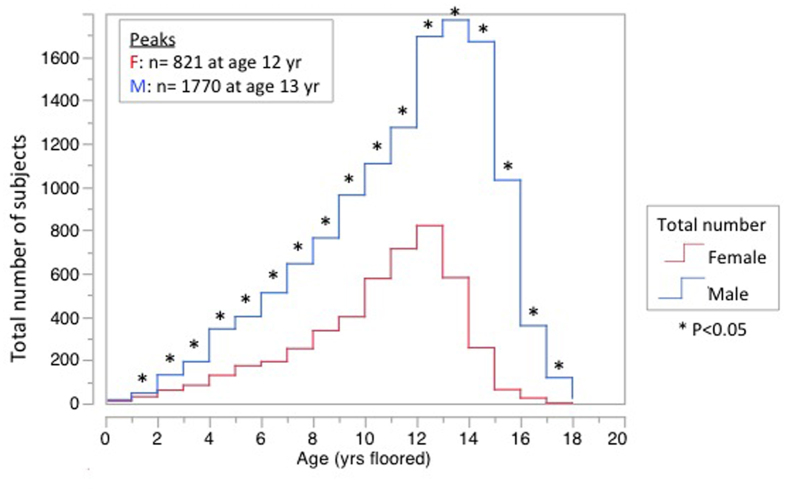
Total number of males and females in the four U.S growth hormone registries by year of age at initiation of treatment for idiopathic short stature. Gender differences for each age were compared by one sample exact binomial test (one-tail) against the null hypothesis that prescription rates were independent of gender.

Males comprised 66% of the 93,736 subjects enrolled in the four U.S. GH registries combined, and 74% of those (18%) treated for the ISS indication (Table 4). The ISS subgroup also differed (P < 0.0001) from the other indications regarding racial/ethnic composition, and neither reflected demographics from U.S. Census data (Table 4). At the time of GH initiation for ISS, females were shorter than males in each of the four registries and in all of the registries combined (mean height Z-score −2.8 ± 0.1 SD vs −2.5 ± 0.1 SD, respectively, P < 0.0001). At GH initiation for ISS, males outnumbered females (P < 0.05) at every year of age except the first year, with the greatest numbers prescribed and the greatest gender difference occurring during the peri-pubertal period (Fig. 3). The median age [interquartile range] at GH initiation was 11 [8–12] years for females and 12 [9–13] years for males.

## Discussion

There were no gender differences in the prevalence of height <−2.25 SD, the ISS threshold, in this large, heterogeneous sample of children and adolescents cared for in a primary care setting. In contrast, GH registries documented that more males than females are treated in the U.S. (2M:1F), especially for the ISS indication (3M:1F). While male predominance among U.S. GH recipients has been described since the first published registry report of GH use in 1985–1987^6^, the current study is unique in combining data across all four U.S. pediatric GH registries, which are usually analyzed and reported separately due to the proprietary nature of the data. A detailed analysis of one of the registries, taking into account the accumulation of FDA approved indications, showed that the almost 2 M:1 F ratio was consistent across the first twenty years of recombinant GH use, possibly related to off label prescriptions^3^.

The greatest numbers of patients started on GH treatment for ISS occurred during the peri-pubertal period for both genders, with the median age at GH initiation of 11 for females and 12 for males. This likely reflects the physiologically earlier onset of puberty in females, and the greater concern about short stature when puberty heralds ultimate growth plate fusion and hence, limited remaining opportunity for potential medical intervention. The maximal gender disparity for GH prescribing occurred in the pubertal age range, raising the possibility that some children were treated with GH for constitutional delay of growth and puberty (CDGP) unnecessarily. However, the number of males prescribed GH for ISS exceeded females for every year of age except the first, suggesting puberty contributes only part of the disparity.

**Figure 4:**
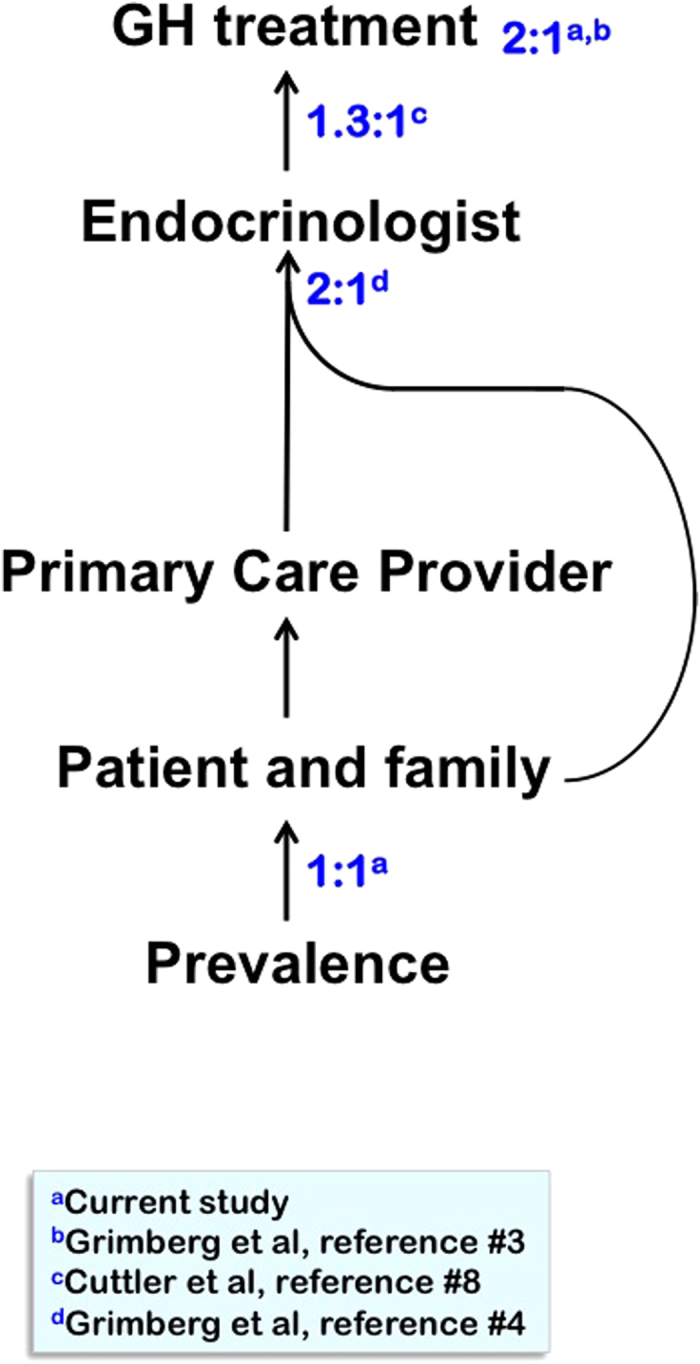
Sequence of events determining who receives pediatric growth hormone treatment. Ratios indicate male:female proportions at the various steps, with their respective references. The 2:1 male predominance among children receiving short stature evaluations at endocrine clinics results from the combination of gender-based referral biases by their primary care providers and gender-based biases of patient-families who directly seek specialist evaluation; the relative contributions of these two sources could not be quantified.

Gender was not a significant predictor of height below the ISS threshold in our primary care population, yet males were 74% of patients treated for ISS and were on average taller at time of GH initiation. What causes this treatment bias (Fig. 4)? GH is prescribed mostly by pediatric endocrinologists^7^. In 1996 and again in 2010, U.S. pediatric endocrinologists, evaluating hypothetical case scenarios in surveys, were more likely to recommend GH treatment for boys than girls in otherwise identical scenarios^8^,^9^. Also, pediatric endocrinologists see about twice as many boys than girls for evaluation of short stature; further, the female patients referred to a pediatric endocrinology center had greater height deficits than the boys and a risk ratio of 2.7 for having an identifiable organic disease^4^. While primary care physicians (PCP) are responsible for specialist referrals, their decisions are influenced by the level of parental concern^7^, and sometimes parents seek specialist care directly. Thus, it is difficult to quantify the relative contributions of parents and PCP to the endocrine referral bias. Many children with short stature do not see a pediatric endocrinologist and are managed by their PCP. In a 3-year study of four urban pediatric primary care practices affiliated with a tertiary pediatric hospital, only 2.8% of children with linear growth faltering saw an endocrinologist, and PCP obtained laboratory tests of the GH axis for twice as many boys (1.8%) than girls (0.9%; P < 0.05) with growth faltering^10^.

Because the height Z-score references were developed for boys and girls separately, it may seem an inevitability to find no gender difference among children with heights <−2.25 SD. However, two factors may alter the gender-based height distributions in a population. The first possibility involves secular trends or other differences between the specific study sample and the CDC reference population upon which the growth charts were based. The second deviation may result potentially from different frequencies of growth-impairing diseases by gender. Similar to a study of two Australian survey populations in 2006–2007, we found evidence for a secular increase in height (mean and median for both genders were greater than zero relative to CDC reference) but no change in the spread of heights across the population (standard deviations of height Z-score distributions in the studies remained 1 SD)^11^. In the Australian study, more boys than girls fell below the CDC and study-specific 1^st^ percentiles, but the actual numbers of children were small (<30 of each gender in each survey)^11^. The authors found no gender difference when the Australian criteria were applied to the National Health and Nutrition Examination Survey (NHANES) of U.S. children in 2005–2006^11^. We likewise found no gender difference in height <−2.25 SD in our population, even though based on the GH registries, one would expect to find more short boys than girls.

The factors we found significantly associated with short stature in our population were consistent with the previous literature. History of premature birth was the strongest predictor of height <−2.25 SD in our logistic regression model even with the analyzed growth data limited to after age 2 years. Increased prevalence of height <−2SD has been reported in Swedish and French cohorts of children born prematurely when measured at age 11 and 5 years, respectively^12^,^13^. Age had the second largest effect on height <−2.25 SD in our population, with OR of 2.66 [2.15–3.28] over the age range of 0.5 to 20 years, likely reflecting the greater opportunity for accruing a height deficit with time. Although males with CDGP frequently seek medical attention, CDGP occurs in adolescents of both genders^14^,^15^ and in our population, males were not significantly shorter than females after age 10 years. Race/ethnicity differences were also found in our model, with Hispanic and Asian children having greater risk and black children having lower risk of height <−2.25 SD than white children, consistent with other studies^16^,^17^,^18^,^19^,^20^,^21^,^22^. Medicaid insurance coverage compared to private insurance was a risk factor for height  < −2.25 SD in our model. Medicaid coverage is often used as a surrogate for lower socioeconomic status and access to healthcare. The detrimental effect of low socioeconomic status on height has been appreciated since 1892^23^ and confirmed in the various NHANES studies^24^,^25^. Finally, BMI Z-score had a strong, but inverse effect in our model of height <−2.25 SD. Poor nutritional intake can stunt statural growth, especially during periods of rapid growth, and nutritional repletion leads to catch-up growth^26^. Conversely, obesity has been linked to accelerated growth and early pubertal development^27^. Secular trends in BMI have been associated with the other factors in our model^28^. Despite complex interactions, the fact that race/ethnicity, Medicaid insurance and BMI Z-score reached significance to P < 0.0001 in our model suggests that they each exerted an independent risk on height <−2.25 SD.

The retrospective nature of this study introduced limitations. Socioeconomic data were not available beyond patient insurance type. To address this, 30,638 subjects were geocoded to extract census-tract data on family structure, maximal household education level, employment and income. The census-tract level variables did not contribute significantly to height <−2.25 SD in our logistic regression model (data not shown), possibly due to variability among residents of a census tract. Socioeconomic factors have been found to influence the medical management of short stature. In an ethnically diverse mid-sized U.S. city, parents seeking evaluation of their child’s short stature by a pediatric endocrinologist were proportionally of higher income and education levels than the surrounding population^29^. Nonetheless, the limitations of retrospective electronic health records (EHR)-based studies would not be expected to alter the gender-based prevalence of height <−2.25 SD in a primary care population.

In conclusion, the prevalence of height below −2.25 SD, the threshold for ISS, was not different by gender in this large, heterogeneous pediatric primary care population despite a male predominance among U.S pediatric GH recipients (74% for ISS). This is the strongest evidence to date supporting the existence of gender-based referral and treatment biases for short stature in U.S. children. Growth failure is a vital sign of child health and should receive equal import for both genders^30^. By focusing on the social aspects of height, whose pressures seem to affect males more than females in U.S. society^31^, the resultant gender-based biases can lead to missed diagnosis of underlying disease in short girls while promoting over-zealous treatment of healthy short boys with an expensive medication.

## Methods

### Subject selection and patient data

All experiments were performed in accordance with relevant guidelines and regulations. The Children’s Hospital of Philadelphia institutional review board approved this study, and determined that the criteria under 45 CFR 46.116(d) were met for waiver of informed consent. The pediatric primary care population was drawn from the 28 practices in Pennsylvania, New Jersey and Delaware affiliated with a tertiary pediatric hospital. The EHR (EpicCare [Epic Systems Inc, Verona, WI]) of all 190,246 patients aged 0.5–20 years who had at least one recorded height or length measurement in 2006 through 2008 were reviewed. Details of patient data extraction methods have been reported previously^5^,^10^ and included: height (or length), weight, BMI, age, gender, race/ethnicity, history of prematurity, insurance provider (private, Medicaid or self-pay), and number of well visits. Practices were categorized as urban or non-urban depending on whether they were located within city population center >100,000.

Height/length, weight and BMI were converted to age- and gender-specific percentiles and Z-scores^5^,^32^. Fidelity of the anthropometric data was optimized by exclusions and manual checks as previously described^5^,^10^. We excluded growth data for low birth weight or premature infants before age 2 years and all biologically implausible heights, defined by the World Health Organization and CDC as beyond −5 or +3 SDs^33^,^34^. Additionally, manual review of the growth charts with outlier BMI Z-scores led to the exclusion of subjects with BMI Z-score below −10 SD or greater than +9 SD as implausible. The resultant study population contained 189,280 subjects.

### Statistical analyses of population data

All analyses were performed with JMP software (SAS Institute, Inc, Cary, NC). Categorical variables were presented as percents and compared by the Pearson χ^2^ test, while continuous variables were presented as mean ± SD and compared by 2-sided *t* test. Logistic regression analyses with effect Wald tests were performed to quantify the relationships between the potential explanatory variables and height <−2.25 SD; model results were presented throughout as partial odds ratios with 95% confidence intervals (C.I.) and χ^2^ P values. Odds ratios provided for the continuous variables were range odds ratios.

### GH registry data

Demographic data were extracted in summer 2012 from the National Cooperative Growth Study database (NCGS; Genentech, South San Francisco, CA), Pfizer International Growth Study database (KIGS; Pfizer Inc., New York, NY), Genetics and Neuroendocrinology of Short Stature International Study (GeNeSIS; Eli Lilly & Company, Indianapolis, IN) and American Norditropin Studies: Web-Enabled Research Program (ANSWER; Novo Nordisk Inc., Princeton, NJ) by methods and collection previously described^35^,^36^,^37^,^38^. Subjects treated for ISS and non-ISS indications were compared by Pearson χ^2^ test. For reference, demographic data from the 2005 and 2011 U.S Census^39^ were provided, contemporaneous with our observation window of the primary care population and the GH registry queries. Mean height Z-scores of males and females at initiation of GH treatment for ISS were compared by student t-test with both Satterthwaite and Welch’s adjustments for unequal variances. Age (years floored) at GH initiation for ISS was tabulated by gender and compared by one sample exact binomial test (one-tail) against the null hypothesis that prescription rates were independent of gender.

## Additional Information

**How to cite this article**: Grimberg, A. *et al.* Gender Bias in U.S. Pediatric Growth Hormone Treatment. *Sci. Rep.* **5**, 11099; doi: 10.1038/srep11099 (2015).

## Figures and Tables

**Table 1:**
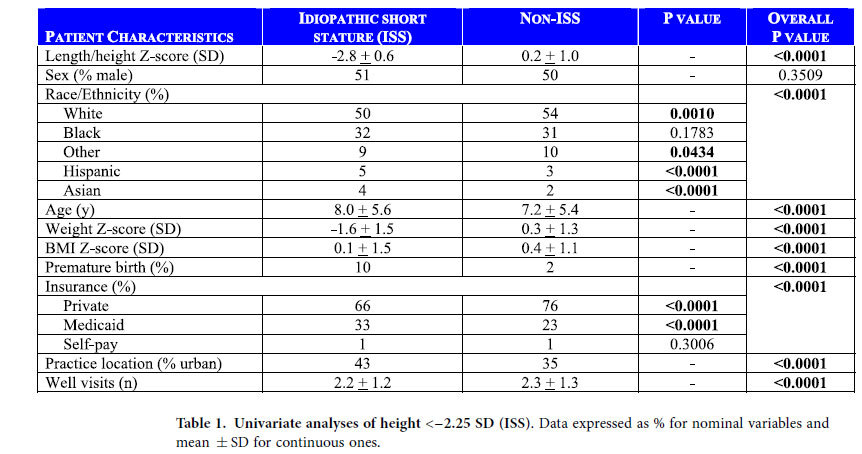
Univariate analyses of height <−2.25 SD (ISS).

**Table 2:**
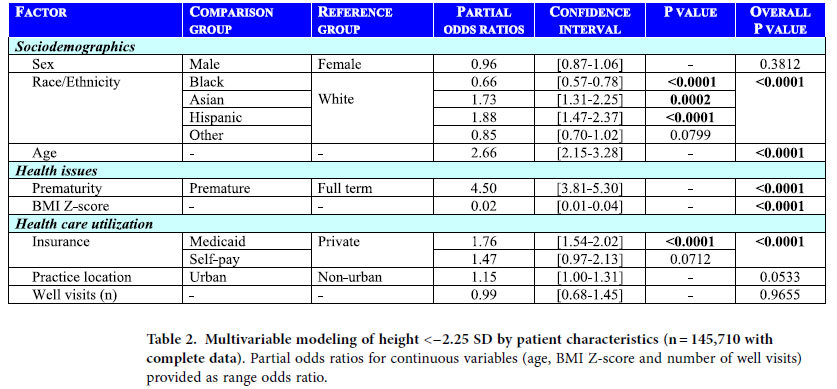
Multivariable modeling of height <−2.25 SD by patient characteristics (n = 145,710 with complete data).

**Table 3:**
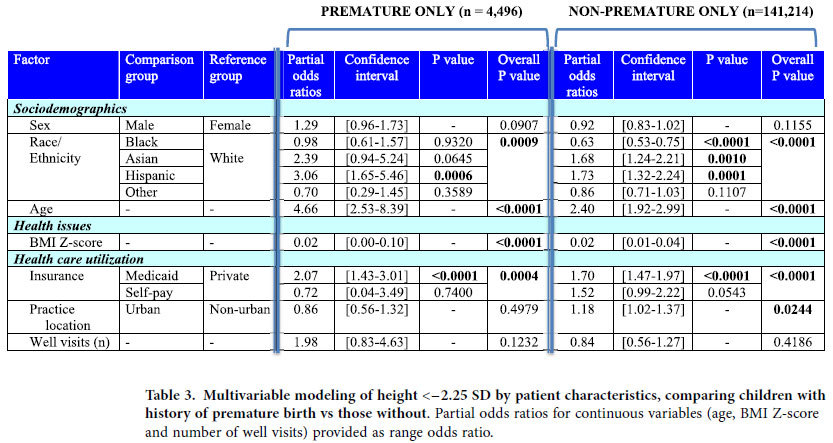
Multivariable modeling of height <−2.25 SD by patient characteristics, comparing children with history of premature birth vs those without.

**Table 4:**
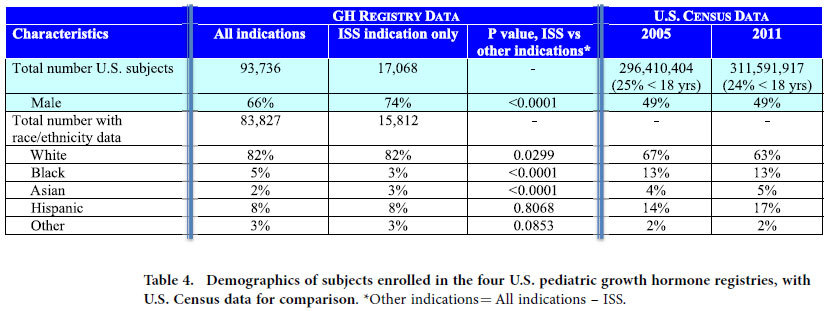
Demographics of subjects enrolled in the four U.S. pediatric growth hormone registries, with U.S. Census data for comparison.
